# The Social Policy of Voluntary Hospitals

**Published:** 1910-10-15

**Authors:** C. S. Loch


### SPECIAL INSTITUTIONAL ARTICLES.

### The Social Policy of Voluntary Hospitals.

By C. S. LOCH, LL.D., D.C.L.

(Read at the First Conference of the British Hospitals Association, Glasgow, September 29 and 30, 1910.)

No people are more grateful than members of the Charity Organisation Society to Hospitals and their staffs for their constant help and advice in cases of illness and distress. To any observant onlooker the advance made by hospitals in the last forty years is evident and remarkable. Within the hospital there has been an advance of administration progressive with the advance of medical science. The nursing system has been greatly developed and in many ways perfected. Convalescent accommodation is used as it never was used before. In relation to agencies outside the hospital also there is an advance. The criticism of out-patient departments by medical men and laymen during the past forty years is having its effect. Hospital almoners are introducing a new discrimination and better methods of general assistance. Hospital finance, thanks largely to Sir Henry Burdett, is better regulated and accounted for. And in London we have the King's Fund, which with the grant of its aids and subsidies is assuming powers of inspection.

Here, then, are indications of a social policy. The questions I desire to submit to your consideration are whether, consistently with these indications, some more definite social policy should not now be developed and accepted by hospitals generally, a policy consistent with the principles of charity and our social and economic knowledge; and, if so, what that policy should be. It is admitted that many changes are being advocated and promoted which may in the future greatly affect the temperament of the people and their attitude towards relief and State aid. There is, therefore, the more reason, I think, for the consideration of this subject now.

Three points may be set down: the hospital in relation to the medical profession and education; the hospital in relation to the Poor Law and medical charities; the hospital in relation to the individual case and its proper assistance in connection with other charities.

### The Hospital in Relation to the Medical Profession and Education.

Broadly, a hospital is a place for the free or part-pay medical and surgical treatment of poor people. The normal transactions between members of the medical profession and their clients are on economic lines, modified by certain social adjustments. The richer pay more relatively for the same or similar work; the poorer pay less by the introduction of the provident or club system in some form, or in reduced fees. Thus, relatively, every hospital in effect makes a breach in the economic method of remuneration, and all the hospitals taken together may make a great breach in it. The offer of the best or very good treatment on a large scale for no payment must draw many claimants. It must also affect many indirectly. What they may get free, there is, they may think, no reason that they should try and provide for themselves or pay for. Some may urge, no doubt, that the mass of the working classes should be treated free. I do not argue that point here; I will do so later. But that a free supply of medical treatment always available must affect the economic supply is evident, if the free supply is in bulk large enough proportionally. In London general practitioners say that the temporary closing of an out-patients' department increases their practice; and undoubtedly in certain areas the free supply is in proportion sufficiently large to affect the economic supply. And so in London generally. In 1905 the out-patients and casualties at the voluntary hospitals alone amounted to 1,859,809. Such a number must affect the conditions of the economic supply of treatment to the industrial classes as a whole; and certainly it is the very strong, and I think justified, conviction of general practitioners that it does so. Hence arise proposals, that are receiving much support, for a revision of the method of medical remuneration and of the system of hospital admissions. In general terms the proposals come to this: that the letter system should disappear; that every patient at a hospital should pay according to his ability; that, in what is called contract work, medical men should be remunerated for their services according to certain standards accepted by the profession locally; that there should be local provident clubs or dispensaries with which the members of the profession should be associated; that in general patients should be sent to the hospitals (I refer more especially here to out-patients) from such dispensaries or from general practitioners; that, I think I may add, medical men who take part in the work of the hospital should be remunerated for their work, for, since a payment would be required from the patient when it is right that he should pay, the staff should also be remunerated relatively at least. Thus the outlying gratuitous relief of the hospital would to a large extent be brought into the economic field of normal payment and remuneration.

The movement in this direction does not come from the medical profession only. It comes also from hospital administrators. Mr. Sydney Holland defines a proper hospital patient as a man who can afford to pay something, but cannot afford to pay for what he is getting;[1] and, as we know, outside the United Kingdom the pay system at hospitals is very much more fully developed than it is here. Here, in general, it can hardly be said to be developed at all. At the sick clubs of doctors and at provident dispensaries the members pay for their treatment; and these are institutions widely spread and sometimes very large; but they, and not the hospitals, are the centres of the pay system. Yet, in the case of hospitals too the pay method is creeping in, as, for instance, for better class patients, at

St. Thomas's, Guy's, and at many special hospitals. Recently also, in London, the Education Committee of the London County Council has undertaken to pay hospitals in order to remunerate additional medical assistants for attending at the out-patient department to cases of school children referred to them and to meet incidental expenses.

In contrast to this policy is that of the supply of free medical relief at the cost of the State. Between this and the other policy it is clear that there can be no compromise. They are diametrically opposed. By one, all patients should pay who could do so at all, according to their means. By the other, the rule should be non-payment, the exception payment. The effect of the latter policy would be to make the hospitals public institutions supported by the State, and the medical men who treat the industrial classes State officials remunerated by salary. If the normal methods of economic exchange, in medicine as in other things, tend to promote foresight and strengthen character, then the adoption of gratuitous medical treatment cannot but weaken the social ability and the manliness of men and injure character—for social competence, manliness, and character depend largely on the power of foresight. It may be argued, in spite of this, that no one who has less than 40s. a week should save or contribute to provident institutions, as all his money should be spent from week to week on himself and his family; yet this argument carries little weight, as against the obvious meaning of a widely noted social fact. Clubs and provident institutions and insurance throughout the country show that people in every grade of life, unless it be the very lowest, desire to provide for themselves and to be independent; and this large class, because, and in so far as, they have this desire, are good spenders, and use their 40s. or less well. The club payment or the saving is indeed only a sign of their power to spend well. It is itself a spending with foresight. A free system which is introduced on the ground that the wages of the day are too low would tend to a less careful spending of those wages and to a deterioration in the peculiarly social virtue of foresight cannot but be socially wrong. To adopt as one's guide in social progress the standard of the lowest classes is to run counter to all social science.

Incidentally, here the question of the teaching of medicine and surgery arises. If the hospital is, in fact, a centre for technical education, for education in the art of healing, it appears to me on that account to have as much right to a grant from the Government for the purpose of teaching as any secondary school, polytechnic, or university. The State has adopted the policy of subsidies in regard to education, and the hospital may fulfil the required conditions for the receipt of a subsidy. But this does not in any way contravene what I have advocated—continuance in the policy of promoting economic exchange, as far as may be, in the supply of medical treatment to the industrial classes.

### The Hospital in Relation to the Poor Law and Medical Charities.

If the remuneration of medical men in supplying medical treatment to the members of the industrial classes, except those unable to pay, should be based on economic exchange, subject to adjustment according to social considerations, it will follow that our medical institutions should be developed further on lines consistent with this principle. There is no doubt that the people have made a great advance in the last forty years. Wages are mostly higher, and necessaries are much cheaper. But there has been a change in temperament also. The standard of comfort is higher; a claim is made to a yet higher standard. It is a question, indeed, whether in all classes there is not a desire to take out of salaries and wages a higher return in comfort than existing salaries and wages can produce. The result must be irritation and discontent, and a demand for the good things for which one cannot pay, but which one thinks oneself aggrieved if one cannot obtain either from the State or from some other source.

Mr. E. W. Morris, the Secretary of the London Hospital, describes well the changes that are taking place in the sick poor. He says that there are, first, a change in character; and secondly, a change in social state:—

> "As to the poor patient's change in character, he by no means looks upon himself as a 'miserable object.' He is probably a member of some trade-union. He joins associations to resist the power of the rich. He is perhaps a Socialist. He has his own member of Parliament. He does not accept favours; he claims rights. His tendency is to claim free healing as he claims free education for his children, and free food and old-age pensions.
>
> "As to the change in social status. The sick poor of our modern hospitals is often a person who is not poor *except to meet sickness*; he cannot possibly afford the heavy expenses of modern methods of healing, whether medical or surgical, so he has gradually drifted to the hospitals, where such skilled attention could be had for the asking. He came at first with some diffidence, but this is now getting somewhat threadbare; he accepts the state of things as he finds it. The necessity for laying by for sickness or of insuring against sickness becomes less and less; he takes the *free* relief in sickness into consideration when making up his annual budget. . . He does not take his blessings as a right; he has simply grown used to the existing state of things and accepts them without question. . . . For his existence hospitals are in themselves largely to blame; they have assisted to make him what he is."

These are the people who to a large extent are using the hospitals. But there is another class—the destitute. The word "destitute" has come to be a Poor Law word, and as such has been used in an ill-defined and most exaggerated manner by the Minority of the members of the Royal Commission. Historically in Poor Law administration it has two meanings. In the case of the able-bodied poor, the Commissioners of 1834 used the word as explanatory of a clause of the Poor Law Act of Queen Elizabeth of 1601. They pointed out that in the case of the able-bodied the Act allowed the setting to work of "all such persons . . . having *no means* to maintain them," that is to say, of destitute persons only. But in the case of the not able-bodied—"the lame, impotent, old, blind, and such other among them being poor and not able to work"—the Act requires that they should have "necessary relief." Thus the word "destitution" and the "having no means to maintain them" are not applicable to them; and the Commissioners, in fact, do not apply the word to them. They call them indigent. But afterwards the word "destitute" was applied without discrimination to all applicants for poor relief, and applied sometimes in the absolute sense of "having no means to maintain them," sometimes according to its second meaning of being in great want. Undoubtedly some general word, such as "destitute," was required to define the claim of the non-ablebodied class. In every country some definition is in force, limiting the class of those who may be entitled to public relief. Thus, in Germany "a poor person who needs help and is unable to work, or only partially able to work, is eligible for public relief, if no one else is both liable for his relief and able to relieve him, or if private charity does not provide for him."[2] Unfortunately in England the word "destitution" has been used in both senses—not in this sense only, but in the other also. In practice, however—in medical relief especially—the definition has been always or usually applied according to its less stringent meaning. "Destitution," in this relation, "has gradually come to mean no more than this, viz. inability to provide whatever medical treatment is necessary"; and Sir Arthur Downes says[3]:—

"The Poor Law has been obliged to provide for the sick on a scale continually advancing with the standard of care for the sick, and, as with medical progress this standard becomes more complete, the more unequal, in the absence of effective organisation, is the condition of the man who tries to provide it for himself."

We have thus both at the hospital and at the Poor Law infirmary—both at the out-patient department and at the dispensary of the district medical officer—a considerable change. The claimant for relief is more exacting—more desirous of free treatment. The standard of medical treatment has risen, and a comparatively small payment for medical treatment is less sufficient to cover its whole cost. This implies the necessity of reorganisation on the part of the members of the profession and on the part of the hospitals, and, as we have seen, on the part of the Poor Law infirmaries, in relation to the profession generally.

But a word more as to the destitute, which I have discriminated and defined above. Mr. Sydney Holland[4] points out that out of 709 patients in the London Hospital, 192 were "to all intents and purposes paupers, *i.e.* no money coming in, no savings, no club, and generally with wife and children to support," and ninety-eight were "on the very verge of pauperism, if not already paupers"—or, in all, 40 per cent. were in this sense destitute.

Miss Roberts, in her report to the Commission on "the overlapping of the work of the Voluntary General Hospitals with that of Poor Law medical relief," states "that the extent to which patients receiving out-treatment at the voluntary general hospitals are actually Poor Law cases is about 11 per cent.," and "that the proportion of hospital patients who may be said to be of a similar class as applicants for medical relief from the parish, is about 38 per cent. (This proportion includes the aforesaid 11 per cent.)"

It should be added that the system of repayment is recognised in the Poor Law. It is recognised, that is, for infirmary patients, who from want of means are least likely to be able to repay. But it is not recognised at the hospitals, where the ability of the patients to contribute is obviously much greater.

All this evidence taken together shows that at a hospital there is a large number of patients altogether above the level of "destitution," using the word broadly; that of these many could and should pay something, in accordance with Mr. Holland's definition of a proper hospital patient—"can afford to pay something," though they "cannot afford to pay for what they are getting"; that many cases in voluntary hospitals are not merely cases of illness but of distress generally also, and that on that ground they should be dealt with by the Poor Law, or public assistance, or by general charity, and cannot be properly dealt with except in that way; that in out-patient departments there is overlapping with the Poor Law to the extent of 11 per cent.; that in the main probably the question is not one of overlapping, *i.e.* two or more authorities dealing at the same time with the same case, but of a bad division of labour; that the tendency of medical institutions, whether they be voluntary hospitals or the Poor Law infirmaries, is to raise the standard of medical treatment, at least for indoor relief, beyond the standard of payment that may be made by the industrial classes; that a division of labour between the voluntary hospital and the Poor Law infirmary should be founded on the lines of partial payment at the hospital and free treatment at the infirmary, or in the reception at the hospital of a class of medical case in which partial payment may fairly be expected, and in the reception at the infirmary of a class of medical case in which payment could not be anticipated as likely to be forthcoming. This division according to social condition may to some extent be found to be reflected in the distinction between acute and chronic cases. Dr. Lauriston Shaw says:—

"All persons ill enough to be confined to bed, and unable to obtain in their own homes sufficient attention to secure a reasonable amount of comfort and a fair prospect of recovery, require treatment either in an infirmary or a hospital. The determination of which of these two hospitals should provide the accommodation should not depend on the social status of the patient, but on the nature of the illness. If the illness require only a moderate amount of medical and nursing skill and attendance it should be treated in the infirmary. If it requires the medical aid of a specialist for its treatment, if it requires the constant attendance of dressers or nurses, or if its observation is likely to lead to important additions to medical knowledge, it should be treated in a voluntary hospital."[5]

This may seem to relegate to the infirmary all the work which would be less attractive to the profession, and to draw the attractive work away almost entirely to the hospitals; and thus to lower the professional status of the work and officers at the former. Properly, the acute case and the specialist work would be connected, one would think, with the teaching centre—now the hospital. But, if, as has long been proposed, the infirmary was also used as a teaching centre, associated with the hospital, this difference would to a large extent be neutralised.

At present, however, the relation of the hospital to the infirmary is far from satisfactory.

"An extraordinary antagonism seems to prevail between the two agencies. Relieving officers are fretful of the constant claims on their time, taken up by application for removal from their local hospitals of the homeless, or of people who do or do not belong to that particular Union. It was said that the wrong class of person is sometimes sent from the hospital to the infirmary, and that the hospital encourages people to demand relief, sometimes giving them open slips addressed to the Relieving Officer." [6]

If these and other difficulties are to be met, there seems, I think, but one way of proceeding, that recommended by the majority of the Royal Commission on the Poor Laws and relief of distress; namely, "that representative Medical Association Committees should be established, to co-ordinate, and, when necessary, supplement the medical institutions of the county or county borough, and to support methods of co-operation with the sanitary authorities and the authorities in charge of voluntary hospitals, to organise an outdoor and provident medical service," etc.

In this way a social policy would be carried out which would, consistently with the interests of the medical profession, place accommodation at hospitals and infirmaries respectively within the reach of patients, who, paying according to their ability, could on medical grounds be referred either to the hospital or to the infirmary. The status of the two institutions would be brought into some kind of equivalence. "Poor Law" cases admitted to the hospital for special treatment might be paid for by the "Poor Law," and "hospital" cases admitted to the infirmary might be transferred to the hospital and maintained by it; and as the case required, admission to one or the other institution would be available from the club or provident dispensary. Thus payment would be made always, as far as it might reasonably be done, and where the illness required it the treatment that could not be paid for would be supplied at either the hospital or the infirmary.

### The Hospital in Relation to the Individual Case and its Proper Assistance.

The third point remains—the hospital in relation to the individual case and its proper assistance in connection with other charities. It is sometimes forgotten, I think, that the whole system of public or voluntary relief is to be judged by the efficacy of the treatment of the individual case. Hospitals, like other charities, have given their relief with regard to sickness, quite irrespective of other causes of distress or other methods of help. This, indeed, is still the rule and not the exception. But a hospital case is not unlike other cases. Properly there should be the same inquiry into facts and causes, the means of assistance and the provision made, with a view to a definite plan being formed and carried out for helping the individual and the family. It is only now that this is being recognised. In no hospital, I believe, is it yet done in all cases and thoroughly. But a great step has been made.

### Hospital Almoners.

In 1892 [7] the Charity Organisation Society, alike to meet the insufficiency of the general assistance forthcoming in the cases of persons who applied to hospitals and to prevent the misuse of hospitals by persons who could provide for themselves, proposed the introduction of the hospital almoners' system. In 1895 it was adopted at the Royal Free Hospital, and carried on for some time as a joint charge on the hospital and the Charity Organisation Society. It is now adopted at some fifteen hospitals in London as well as in some hospitals in provincial towns. It is distinctive in two ways. A high standard of training and competence was set up almost at the outset. The almoner has to work at Charity Organisation committees to learn the principles and methods of charity; to work at a hospital under an almoner, in order to learn the special conditions of hospital work; and to study social economics and administration at the School of Sociology. The training usually lasts for from a year to eighteen months. The plan has been to equip the almoner for her duties by practice and by a correlative study of social facts and theory, and at the same time to claim for the almoner a standard of salary consistent with the skill and ability of a trained officer. The other distinctive point was this. The hospital almoner regularised the out-patient work. She took down the cases, thought out plans, indexed the cases, but, except in the case of comparatively small funds at some hospitals, she had no relief directly at her disposal. Accordingly she became an organising centre. She supplemented what the hospital was doing by what the charities might do. In this way she ensured as far as possible that cases dealt with at the hospital were aided, so that the medical treatment might become effective. If that hospital almoner is recognised by the council of the Charity Organisation Society, the Society is ready to make special supplementary inquiries for her, and from the district committees of the Society constant help is forthcoming for a very large number of cases sent to them. A whole series of new connections has thus been attached to hospital relief in order to make it effectual. There is now in London an Hospital Almoners' Council for the supply and education of almoners, which will, I hope, very shortly be further developed, and will receive the support of the hospitals generally and of the public. The reports of almoners show how useful their work is.[8] If it is properly done, this must be so. No single institution is sufficient, as a rule, for doing all that a "case" needs. The organisation of charity consists of a true interpretation of the needs of the individual, and such a combination of methods as will meet his needs, prevent his distress in future, as far as possible, and make him independent. This is the aim.

I would submit to you then the outlines of a social policy for hospitals, under the three divisions of this paper. This policy would lead, I believe, as experience has shown, to a better remuneration of medical men; to a better division of labour among institutions for the sick; and to a very serviceable association in charity. It would help the individual physically and morally, as, I believe, no other policy can do, for it is based on the principle of a proper treatment of the individual, not as a social item but as the member of a family, and as the member of a society which would have all its members hale and independent.

---

[1] Evidence, Royal Commission on the Poor Law. Vol. iii., *Q.* 32799.
[2] Report of the Royal Commission on the Poor-laws. Part VII., § 155. The Commission suggested the use of the word "necessitous" to avoid misunderstanding.
[3] *Ibid.* Part V., § 13.
[4] *Ibid.* Evidence, Vol. III., p. 590.
[5] *Ibid.* Evidence of Dr. Lauriston E. Shaw. III., 33118.
[6] Report. Miss Roberts, p. 13.
[7] *Ibid.* Montifiore, Vol. III., p. 24. 3505.
[8] Evidence of Miss Helen J. Nussey. *Ibid.* Evidence, Vol. III. 32917.

### DISCUSSION.

Mr. James Cunningham, J.P. : Dr. Loch confines himself practically to a statement of facts which we all deplore, and to recommendations with which we are all practically agreed. In dividing his paper into its three heads he deals first with the voluntary hospital *versus* payment of medical men, and in many cases medical men acting under the Poor Law relief are not properly remunerated for the work that they have to do. They are labouring under very difficult and very trying circumstances in most cases, and we are all agreed that there should be better pay for these gentlemen. He refers also to the State provision of hospitals. I think it is well that we should call very strong attention to the State provision for everything, which seems to be the tendency of the day, and will lead, I am afraid, to the encouragement of thriftlessness and a lack of independence. Dr. Loch very wisely emphasises that in some of the remarks in his paper. The subject of clubs was also referred to, and I think that we have in Scotland a good many of these benefit associations, such as the Foresters and friendly societies of various kinds, which not only give medical assistance, but also relief of another kind. I understand that these people pay simply for aid in the case of sickness, and I think a development of that might be very good if we could compel it; but I don't think that the people who are under our Poor Law are the class of people who will join such clubs unless they are compelled. We don't find that the trade unionist and the decent working man, as a rule, comes under our Poor Law or requires assistance in our Poor Law hospitals, but it is the thriftless and the lazy and that class of people to a large extent who are using our hospitals under the Poor Law relief. Then Dr. Loch, in another branch of his paper, suggests partial payment. There are a great many people using these voluntary hospitals who should not get the benefits free. It is quite right that they should get the use of them, but they should be made to pay something for that. The general idea seems to be that because they contribute a few pence per annum through the factories or their place of employment, therefore they are entitled to get all that possibly can be given, and they have no idea of paying anything for it. I will go further, and say that in connection with our City Health Department our hospitals for infectious diseases should not be free to every ratepayer, but that there should be a consideration whether the men who are getting the advantage of these hospitals should not be made to pay something for the special treatment beyond the mere question of payment of rates. Anyone who is entitled to Poor Law relief is entitled to the hospital accommodation that we can give them. With regard to the last division, of hospital *versus* individuals, I like Dr. Loch's suggestion very much that there should be almoners in connection with these hospitals—that is, that a working man who is only requiring some financial assistance because of his illness should not be brought under the Poor Law and not have the stigma of getting Poor Law assistance. We have abundance of charities in all our great centres that would help this man over his temporary difficulties without having him in receipt of Poor Law relief. We have many charities which find difficulty, I believe, in disposing properly of all their funds; and if we had the proper organisation there would be no difficulty in helping this man in his temporary difficulty and so keeping him from coming under the Poor Law. I think it is right that these people should not have the stigma of Poor Law assistance.

### Individualism and State Aid.

Professor Jones : There are a number of things known to people engaged in philosophy as pre-suppositions—things taken for granted—which I think it would be well to have examined. I think that payment according to ability is a thing that must approve itself to everyone. There is also a payment for proper service to men engaged in hospitals, and I think that is an admirable thing. We all know that there is a transformation going on in the great social changes to which Dr. Loch has referred. Some such change is the organisation of labour; and then there are facilities for communication, enabling doctors in the country to send cases to hospitals which originally they had to treat themselves, so that the hospitals, or the infirmaries, as we say here, come to be the centres for the greatest medical skill that a great city can command. Well, if that is so, then these should be open to rich as to poor. I, for my part, would like to be able in cases of illness in my own family to send them where I knew that they would get the best medical skill and surgical skill that the city can afford, and I would like to pay for that. Is not that a right thing? It seems to me that a great expansion in the use of infirmaries would be to have a paying wing. There is no reason at all why we should not imitate what the Americans do and institute on a large scale a free wing. We have a certain amount of medical skill in the community, and the question is to employ the organising powers which the medical profession has shown in so many ways so as to secure the greatest possible use of it most economically. I should like to raise the question of the independence of the character of the individual as regards the State. I do not disregard the ethical question, but I would deny frankly the pre-supposition that State-aid implies impoverisation. I am a State servant myself, and as far as I know I am as independent as my neighbours. Corruption is not to be taken for granted. We cannot assume that State aid from the *a priori* point of view is a thing that is to be condemned. It is a thing that is to be used. The *a priori* assumption that the degradation of character is going on owing to State aid is one of the pre-suppositions that I would ask you to examine. So far as I know, banks for the working men show as good results as ever they did, and all this about State aid I would ask you not to assume to be true as a matter of fact. The question is how far is it open for us to extend our infirmaries in such a way as to enable our citizens to make use of them if they desire, and also to pay for them. It seems to me that the development of this is a matter which would benefit the community greatly.

### The Condition in London.

Mr. Conrad W. Thies (Hon. Treasurer of the Association) : It may be in the memory of those who have followed this hospital question for some years past that a Commission of the House of Lords sat for three years making an inquiry into questions of hospital relief and hospital education in London. That Commission made a very valuable report and certain recommendations, and I think I may say that until this day they have been practically a dead letter. One outcome of the recommendations of that Commission was that in London an attempt was made to form a kind of Hospital Council, the main recommendation of the Commission having been that such a Board should be officially established and would bring together all the different agencies for the relief of the sick. Throughout this country we have a method of moving on from one precedent to another very slowly and endeavouring to take hold of the whole problem and deal with it as a question to be solved for the day and generation. I speak, of course, more intimately of the conditions of hospital relief in London than in those of Scotland. I am well aware that this city of Glasgow has set an example to the whole of Great Britain with reference to the thoroughness with which it has dealt with the problems of a great city community, and here I believe you are nearer to the solution from a local point of view than perhaps we are in London. We have the fact that there are twelve medical schools competing with each other, and we have about 120 hospitals competing with each other—competing not only for funds, which is a daily and terrible responsibility on those who are running these institutions, but competing for patients and competing in every possible way, and instead of working together for one common end they are overlapping. In all these circumstances one naturally looks around to see what other countries are doing in this matter. It has been my privilege to visit hospitals in every part of the world. I spent two months visiting hospitals on the other side of the Atlantic, and I visited them in Australia and Egypt and India. I do not think there is any part of the world where the English language is spoken where I have not had the opportunity of visiting and studying the problem, and I have come to the conclusion that the only people that have grappled with this problem in a serious, business, and scientific manner are the Germans and the Swiss. In America vast sums of money have been expended to try and bring this question to an issue. They have there an association which we are only beginning to copy, where they gather together 1,000 delegates who discuss day after day for a week together all the various problems arising out of this question—the relief of the sick and the education of the rising generation of medical men. In Germany the care of the sick is looked upon as a part of the duty of the public. In the London County Council Education Committee they are already beginning to subsidise the voluntary hospitals in order that children in the early stages of disease may be watched and frequently attended to so that prevention may be better than leaving the children to become chronic invalids. But out of all this chaos in London I do not see any way out of the difficulty at present, unless we can get Parliamentary powers to deal with the whole problem. The interests are so diverse and so conflicting that I do not see any daylight, as it were. There are many movements that have been made to bring this to a direct issue. The establishment in London of the King Edward Hospital Fund has been a great financial success, but to my mind it has only staved off the difficulty, and has not removed the cause of that difficulty. It has brought together for the support of the voluntary hospitals in London a vast amount of money which has saved many of the institutions from bankruptcy; and yet I can assure you that at the present time there is hardly a voluntary hospital in London that is not faced with financial difficulty, in spite of the fact that a large amount of money has been received. On the other hand, we have in London provided out of the rates accommodation for some 25,000 or 26,000 beds, while the voluntary hospitals in London number 10,000 beds, and in addition to these they provide for fever cases and lunatics over 30,000, all paid for out of the rates, so that you have in London out of something like 65,000 beds for the sick, 55,000 being paid for already out of the rates; and yet, in the face of that fact, I think the idea is that the whole problem should be dealt with, and dealt with so that it becomes a municipal institution, and you are brought face to face with the objection that you destroy the voluntary principle.

### The German Example.

Now, what is the result of the principle as it is carried out at present? I have heard it stated by a Board of Guardians that you have no right to deal with the destitute pauper, and we are told that we are not to deal with people who are in receipt of a certain wage, and then you get a section of the community who are between the pauper, on the one hand, and those who are able to look after themselves in some way. The result is that the very poor in London have more than is required for medical relief. Many of these infirmaries are of a modern character and well equipped and well officered. Then you have the great section of the people on whom rests the burden—the great middle-class, who have no means of meeting extraordinary expenses. If you take a clerk with a salary of about £200 or £250 a year to support a family, you have a case where an illness might cost him £100 for some member of his family, and he has been crippled for years by that enormous strain upon his resources. Then there are the wealthy class who can afford to pay and are only accommodated at great cost in numerous nursing homes. Now, what is the result in Germany? I visited hospitals in Berlin, in Hamburg, and Hanover, but the ideal hospital that we should endeavour to imitate is the great hospital at Eppendorf, on the outskirts of Hamburg. They have the destitute patient, who is paid for by the district where he resides at so much per day—about 1s. 6d. a day—and they have a class above that of working people, who are compelled by law to contribute something to a trade insurance against sickness. Their employers are also compelled to contribute, and out of that the expense at the hospital is paid. Then, there is a better class—the middle class—who can afford to pay, and who are assessed according to their means. Above that you have the well-to-do class of people—the people who have no opportunity of using the wonderful resources of our great hospitals. I personally feel greatly indebted to Dr. Loch for his paper, and I do not think that the British Hospitals Association could have had a finer exposition of the general principle and general needs than have been stated in this paper, and I am sure that we shall get some ultimate benefit from it.

Dr. Loch (in reply) : Mr. Chairman, ladies and gentlemen,—I think that we are greatly indebted to my friend Mr. Thies, who has such a knowledge of hospitals, and for the suggestion thrown out by Professor Jones. Now, the general question raised, as it were, behind this paper as a pre-supposition, is whether too much stress has been laid upon voluntary intervention as against State intervention, and whether as a consequence the proposals made here which are applicable on this basis would not be applicable on another basis of which the State took the larger part in starting hospitals. I presume the pre-supposition is something like this : it is that, in the main, on the voluntary system we have to throw back on the individual the support of himself or he is taken away from us, and the contention is that that would not apply to State aid; but I should think that the evidence in regard to such a pre-supposition shows that that dilemma equally applies to State aid and voluntary aid, so that I do not see a great breach between myself and Professor Jones. He would say that the State should do more, and I have not taken the question of what the State should do, but rather for a policy whatever happens. Now Mr. Thies says, quite rightly, that in Germany there is an organised system, and practically the basis of that is the Poor Law and a large contributory insurance for medical relief—contributory insurance coupled with two or three other insurances such as old-age pensions. I feel that the members of the Royal Commission should have made a scheme which would be based upon the pre-supposition that there should be compulsory insurance throughout England. We have suggested what I think is a method of observation. The preliminary step is to bring all the bodies together. You may say that thereafter there may be a system of medical insurance on contributory State lines. I am not against contributory insurance, and it is quite possible that we may have State provision of hospitals in new directions. On this basis that is quite possible, and what would happen would be that the voluntary hospitals would find their place in this continually altered grouping, but at present we do not recognise them as a single group. Our first business is to adopt a clear policy which is good for the community and ethically sound, which will pay the profession better for its work and form a true unit from the point of view of organisation. I think Mr. Thies is right in saying we may have statutory intervention, but the statutory intervention may come in various ways. As he says, we do things very slowly in England, and therefore we are liable to make a mistake if we rush out theoretically to anything to which Society is not ready to attach support. The conclusion I therefore put before you is this : On the whole, is the social policy of taking a full account of possible development a sound line to take? I hold that it is sound primarily for this purpose. Naturally a man is shown by the evidence to be inclined to be independent and pay for himself. I should do nothing to weaken that, and I should take this as a prime factor, whether we adopt the State system or lay stress on the voluntary system. I feel that in many ways Scotland is very different from England, and I would not press English opinion on a Scotch audience. All I ask is that if you do not approve of the social policy as here promulgated and settled in your pre-suppositions, do not let us "haver." We have been year after year in a kind of unprogressive mood, subject to the tidal movement of the day, and what we want is a clear advance in some policy which does not commit oneself, but which will produce organisation, and the organisation of individual treatment of all individual cases is what we want.

